# Prevalence of Positive Screening of Sleep-Disordered Breathing Among Children and Adolescents in Orthodontic Settings: A Systematic Review

**DOI:** 10.3390/jcm15020802

**Published:** 2026-01-19

**Authors:** Maurizio Ledda, Chiara Pili, Silvia Mura, Eric Battista, Teresa Cobo, Alessio Verdecchia, Enrico Spinas

**Affiliations:** 1 Department of Surgical Sciences, Postgraduate School in Orthodontics, University of Cagliari, 09124 Cagliari, Italy; maurizioledda93@gmail.com (M.L.); chiarapili01@gmail.com (C.P.); silviamura3@gmail.com (S.M.); eric@eb-dental.co.uk (E.B.); 2 Orthodontics Division, Instituto Asturiano de Odontología, Universidad de Oviedo, 33006 Oviedo, Spain; dracobo@iaodontologia.com

**Keywords:** sleep disordered breathing, obstructive sleep apnea, orthodontics, screening, sleep questionnaires, pediatric sleep apnea

## Abstract

**Background/Objectives**: Sleep-Disordered Breathing (SDB) in children is closely associated with craniofacial growth and orthodontic conditions. Early identification of SDB risk in orthodontic populations is crucial, yet evidence remains fragmented. This systematic review aimed to summarize the prevalence of high SDB risk in pediatric orthodontic patients assessed through validated questionnaires. **Methods**: A systematic search was conducted across PubMed, Scopus, Web of Science, Cochrane Library and Embase following PRISMA guidelines. Inclusion criteria comprised analytical cross-sectional studies assessing SDB risk in children undergoing or seeking orthodontic treatment, using validated questionnaires such as the Pediatric Sleep Questionnaire (PSQ), OSA-18, or Sleep Clinical Record (SCR). The methodological quality of the included studies was assessed using the “JBI Critical Appraisal Checklist for Studies Reporting Prevalence Data”. The certainty of the evidence was additionally evaluated using the GRADE approach. **Results**: Twelve studies published between 2011 and 2025 met the inclusion criteria, totaling 3737 participants. Across studies, the mean prevalence of high SDB risk ranged from a minimum of 1.2% to a maximum of 69%, with consistently higher values in populations exhibiting malocclusions, oral breathing patterns, or craniofacial risk markers. All studies clearly described their populations and used validated screening tools, resulting in moderate overall quality. **Conclusions**: Pediatric orthodontic populations demonstrate a substantial prevalence of high SDB risk, suggesting that orthodontists should systematically incorporate validated questionnaires into routine screening. The evidence base, although consistent, remains limited by methodological weaknesses. Further well-designed studies are needed to clarify causal relationships between craniofacial development and SDB.

## 1. Introduction

Sleep-disordered breathing (SDB) includes a spectrum of abnormal respiratory patterns during sleep, ranging from primary snoring to obstructive sleep apnea (OSA). Within this continuum, OSA represents the most clinically relevant condition and involves recurrent episodes of partial (hypopneas) or complete (apneas) upper airway obstruction during sleep. Clinicians diagnose OSA through overnight polysomnography (PSG), which remains the gold standard for evaluating sleep-related breathing disorders. Clinicians quantify disease severity using the Apnea–Hypopnea Index (AHI), defined as the number of apneas and hypopneas per hour of sleep, and the Respiratory Disturbance Index (RDI), which also accounts for respiratory effort–related arousals [1,2,3]. According to the pediatric classification, an AHI ≥ 1 is sufficient to define the presence of OSA.

The mechanisms leading to upper airway collapse are complex and multifactorial. They include structural narrowing of the pharyngeal airway, obesity, altered muscle function of the upper airway, pharyngeal neuropathy, and nocturnal fluid shifts toward the neck region [4,5]. Beyond the disruption of normal sleep architecture, SDB has been associated with excessive daytime sleepiness, cognitive and behavioral deficits, and increased cardiovascular and metabolic risk [6].

The International Classification of Sleep Disorders identifies OSA as one of the major sleep-related breathing disorders. The 2023 revision emphasizes the integration of pediatric and adult criteria to ensure consistent diagnostic approaches [7]. Despite advances in diagnostic techniques, PSG remains resource-intensive, requiring specialized facilities and trained personnel, and is therefore not feasible as a mass screening tool. For this reason, several validated questionnaires have been developed to identify individuals at risk of OSA. Commonly used instruments include the Berlin Questionnaire, the STOP-Bang, and the Epworth Sleepiness Scale (ESS), which have demonstrated variable diagnostic accuracy across different populations [8,9].

Pediatric OSA presents distinctive clinical and pathophysiological features compared with adults. The primary etiological factor in pediatric OSA is adenotonsillar hypertrophy, though other risk factors include obesity, craniofacial anomalies, neuromuscular disorders, allergic rhinitis, asthma, and premature birth [10,11]. Epidemiological studies estimate the prevalence of primary snoring between 8 and 27%, and OSA between 1 and 5%, with a peak incidence between 2 and 8 years of age [3]. These data highlight the clinical relevance of early detection, as untreated OSA in children may lead to growth retardation, learning difficulties, and behavioral problems.

Among pediatric screening tools, the Pediatric Sleep Questionnaire (PSQ), developed by Chervin et al., is one of the most widely used and validated instruments. It assesses snoring, breathing difficulties, mouth breathing, daytime sleepiness, and behavioral aspects such as inattention or hyperactivity [12,13]. Other questionnaires, such as the STOP-Bang [14,15] and the Sleep Clinical Record (SCR) [16], have been proposed as complementary or alternative screening tools. However, differences in study design, scoring thresholds, and validation samples contribute to variable sensitivity and specificity, limiting their universal application.

Dentists and orthodontists play an increasingly important role in the early recognition of sleep-disordered breathing. During routine dental examinations, clinicians can detect morphological and functional indicators such as snoring, mouth breathing, excessive overjet, or mandibular retrognathia. Orthodontic treatment, particularly rapid maxillary expansion (RME) and mandibular advancement devices (MADs), has demonstrated beneficial effects in improving upper airway volume and reducing apnea–hypopnea events [17,18,19].

Given the growing recognition of the multifactorial nature of SDB and the need for early identification of at-risk individuals, reliable and accessible screening tools are essential for both clinical and research purposes. In this context, several questionnaires have been developed and validated to detect children at risk for SDB based on parental reports and observable symptoms. However, variability in their design, scoring systems, and diagnostic accuracy has limited their comparability and widespread implementation. Yet, despite growing interest in the field, there still appears to be no review that clearly outlines the prevalence of positive screening for SDB specifically within orthodontic settings, leaving this area only partially explored.

Therefore, this systematic review was conducted to evaluate the screening for SDB through scientifically validated questionnaires, with the aim of summarizing the available evidence on their application in pediatric and orthodontic populations, estimating the prevalence of high-risk cases, and identifying craniofacial and demographic factors associated with increased SDB susceptibility.

## 2. Materials and Methods

### 2.1. Protocol and Registration

The present systematic review was performed in accordance with the statement of Preferred Reporting Items for Systematic Reviews and Meta-Analyses (PRISMA). (Supplementary Table S1) [20]. The protocol was registered in PROSPERO, the international prospective register of systematic reviews, on 27 September 2025, under the number CRD420251156701.

### 2.2. Search Strategy

The search covered all articles published up to 20 October 2025 reporting the prevalence of risk of SDB among orthodontic populations and used PubMed, Web of Science, Scopus, Embase, and the Cochrane Library. The grey literature search included the OpenGrey database to ensure comprehensive coverage of relevant studies. No language restrictions or limits on publication date were applied during the literature search. Table 1 reports the detailed search strategies for each database.

Detailed descriptions of the search conducted in the five selected databases: PubMed, Scopus, Web of Science, Embase, and the Cochrane Central Register of Controlled Trials (CENTRAL). The search was customized to each database.

### 2.3. Eligibility Criteria

To define the parameters that the selected studies must respect, we define, according to the criteria of PICO [21], the question shown in Table 2.

The inclusion criteria:

- RCTs, observational studies.
- Studies that involve children and adolescent patients, aged 4 to 18 years
- Patients under orthodontic therapy or patients seeking orthodontic treatment
- Studies reporting screening for SDB risk were performed using scientifically validated questionnaires
- Studies reporting the percentage of high-risk patients and low-risk patients.

The exclusion criteria:

- Case reports, reviews, meta-analyses, case series, dossiers, reports.
- In vitro or animal studies.
- Adult patients (over 18 years old) or, in the case of studies including multiple patient groups, articles where it is not possible to extract data referring specifically to the group of interest;
- Patients who have already completed orthodontic treatment or therapies for SDB at the time of screening;
- Articles included in the screening of patients with pathological dentoskeletal anomalies;
- Screenings are performed in the general pediatric population.

### 2.4. Data Collection

Two authors (C.P. and M.L.) independently carried out the research process first, followed by the screening of the obtained results. To assess the level of agreement among the reviewers, Cohen’s kappa coefficient [22] was calculated. In case of disagreement during the data extraction process, a third reviewer (A.V.) participated in the discussion to reach a decision. The analysis showed a substantial agreement among the authors (Cohen’s kappa: 0.66).

The selection process was performed in two stages: first, all potentially eligible studies were screened by reading titles and abstracts. Second screening was performed according to predefined inclusion and exclusion criteria by full-text analysis of the articles. The collected data were organized into thematic sections, providing a clear and comprehensive overview of each selected study.

- General characteristics. This section covers the essential information of each study: author, year of publication, country, study design, research setting and main conclusions.
- Data about the primary outcome: sample size, gender, type of questionnaire used, percentage of patients at high risk and low risk of SDB.
- Data about the secondary outcome: Questionnaire-based variables significantly associated with an increased risk of SDB, and demographic correlates of increased SDB risk.

### 2.5. Quality Assessment

The methodological quality of the included studies was evaluated using the Joanna Briggs Institute (JBI) Critical Appraisal Checklist for Studies Reporting Prevalence Data [23]. This tool is specifically designed to assess the internal validity of prevalence research and examines nine domains that collectively determine the reliability and applicability of the reported estimates. The checklist evaluates: (1) the appropriateness of the sample frame (2), the sampling method, (3) sample size adequacy, (4) the description of study subjects and setting; (5) coverage of the identified sample; (6) the validity of the condition measurement, (7) measurement consistency, (8) the management of statistical analysis and (9) the response rate and its handling. Each domain was rated as “yes”, “no”, “unclear”, or “not applicable”, based on the information provided in the original articles. Two reviewers (M.L., A.V.) independently assessed each study, and disagreements were resolved through discussion and consensus. Discrepancies were resolved through discussion or, if needed, by consulting a third author (E.S.).

The overall certainty of evidence was further appraised using the GRADE (Grading of Recommendations Assessment, Development and Evaluation) methodology [24]. This system grades evidence as high, moderate, low, or very low, based on risk of bias, inconsistency, indirectness, imprecision, and potential publication bias. GRADE was applied to prevalence outcomes and cross-sectional associations to provide a structured assessment of the certainty of evidence, acknowledging the inherent limitations of observational designs and without implying causal inference.

## 3. Results

### 3.1. Screening and Selection Process

The search strategy yielded a total of 2057 articles through the consultation of five major databases: Embase (700 records), Web of Science (177), Scopus (316), Cochrane Library (52), and PubMed (812). Duplicates were initially removed using Zotero software 7.013 (740 records), followed by a manual search that excluded an additional 9 duplicates.

The remaining 1308 articles were screened based on title and abstract, resulting in 22 studies selected for full-text assessment. 10 articles were excluded during the selection process for the following reasons: two studies involved non-orthodontic populations; one study was ongoing and therefore not yet completed; one focused on screening directed at orthodontists rather than patients; three included patients outside the selected age range; and three did not report a clearly defined primary outcome.

**Figure 1:**
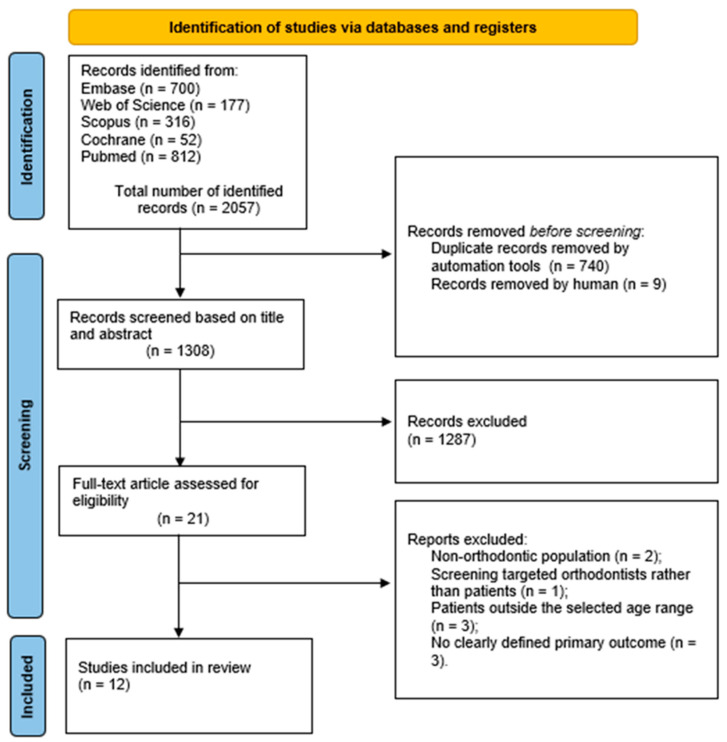
PRISMA flowchart, flow diagram of the performed search.

At the conclusion of the selection process, 12 studies met the predefined inclusion and exclusion criteria. The flowchart of the screening process, in accordance with the PRISMA statement, is presented in Figure 1.

### 3.2. General Characteristics of the Selected Studies

This systematic review includes articles from 2011 to 2025. Although randomized controlled trials were considered eligible a priori, no RCTs meeting the inclusion criteria were identified; all included studies were cross-sectional. All studies adopted a cross-sectional design, with one study (Choong et al., 2022 [25]) conducted across multiple university centers. In terms of geographical distribution, the studies cover multiple world regions. Early contributions originated from Canada (Tsuda et al., 2011 [26]) and the United States (Rohra et al., 2018 [27]), while more recent work expanded to Saudi Arabia (Al Ehaideb et al., 2021 [28]), Malaysia (Choong et al., 2022 [25]), Australia (Wellham et al., 2023 [29]), Israel (Orbach et al., 2023 [30]), Brazil (Jost et al., 2024 [31]), Pakistan (Kanwal et al., 2025 [32]), and European centers in Denmark and Italy (Niu et al., 2025 [33]; Nucci et al., 2025 [34]; Storari et al., 2025 [35]). Regarding the clinical setting, most studies were carried out in university or teaching orthodontic clinics, such as those at the University of British Columbia, Case Western Reserve University, and various European and South American dental schools. Others were based in hospital departments or private orthodontic practices, sometimes combining multiple sites within the same study (e.g., Abtahi et al., 2020 [36], involving both university and private practices; Choong et al., 2022 [25], conducted as a multicenter project across three Malaysian universities). Table 3 summarizes the general characteristics of the twelve studies included in this systematic review.

### 3.3. The Primary Outcome

#### 3.3.1. Sample Size, Age and Gender

Across the twelve studies, sample sizes ranged widely, from a minimum of 48 to a maximum of 1209 participants, yielding a cumulative sample of 3737 children and adolescents. The mean age of patients ranged from 8.1 years in Storari (2025) [35] to 14.4 years in Rohra (2018) [27]. Each study specified both the mean age and the age range of the included patients. The weighted mean age across all studies with available data was approximately 11.8 years. All studies, with the exception of Choong et al. (2022) [25], reported the gender distribution of participants. The proportion of male and female patients varied considerably, from parity in Kanwal [32], near parity in Tsuda [26] and Rohra [27], to a more marked predominance of females in Niu (62%) [33]. Overall, among the 3737 patients for whom sex was reported, 51% were female.

#### 3.3.2. Type of Questionnaire

Most of the included studies [27,28,29,30,31,32,33,34,36] used the Pediatric Sleep Questionnaire to assess sleep-disordered breathing; one study by Tsuda [26] employed the OSA-18, another by Choong [25] used the ESS-CHAD, and a third by Storari [35] applied the SCR questionnaire.

#### 3.3.3. SDB Prevalence

The proportion of participants classified as “high risk for SDB” demonstrated substantial heterogeneity, ranging from 1.2% (Tsuda et al., [26] using OSA-18) to 69% (Storari et al., [35] using the SCR). Conversely, low-risk prevalence ranged from 31% to 98.8%.

Overall, 3737 patients were included, with a mean age of 11.7 years.

Table 4 describes the primary outcome of the studies included in this systematic review.

### 3.4. Secondary Outcome

Across studies using the PSQ, several questionnaire items were statistically associated with a screen-positive classification for sleep-disordered breathing. These included mouth breathing and nocturnal breathing-related symptoms (snoring, breathing pauses, and labored breathing), as reported by Al Ehaideb et al. [28]. Allergic symptoms were also associated with screen-positive status in the PSQ-based study by Kanwal et al. [32]. In addition, questionnaire items related to inattention/hyperactivity and enuresis showed statistically significant associations with screen-positive classification in the study by Abtahi et al. [36].

In the study employing the Sleep Clinical Record (SCR), mouth breathing and specific craniofacial features documented within the clinical scoring system, including a narrow or high palatal vault, were associated with screen-positive classification [35].

No statistically significant questionnaire-item associations were reported in the studies using the OSA-18 or the ESS-CHAD [25,26]. Table 5 summarizes the questionnaire-based variables that were statistically associated with screen-positive classification across the included studies (*p* ≤ 0.05)

In addition to questionnaire items, some demographic factors emerged as significant. Male gender was associated with a higher risk in four studies [28,29,30,34]; younger age (especially preadolescent groups) was a significant factor in two cohorts [31,34]. Table 6 summarizes demographic factors associated with positive SDB screening.

### 3.5. Risk of Bias Assessment

Overall, the 12 included studies demonstrated a generally consistent methodological profile across the nine domains of the JBI Critical Appraisal Checklist for Studies Reporting Prevalence Data. D1 was judged as Yes in all studies, as the sampling frames were clearly defined and appropriate for the target population, namely children and adolescents seeking or undergoing orthodontic evaluation or treatment. D2 was also rated as Yes across all studies, since participants were recruited using acceptable methods within orthodontic clinical settings, typically involving consecutive or convenience sampling aligned with the stated research objectives. D3 showed greater variability. Studies with larger cohorts and clearly reported sample size justifications were rated as Yes, whereas studies with limited sample sizes or insufficient reporting regarding sample adequacy were rated as Unclear or No. D4 received a Yes judgment for all included studies, as participant characteristics, age ranges, clinical settings, and inclusion criteria were consistently and transparently described. D5 was predominantly rated as Unclear. In most studies, it was not possible to determine whether the analyzed sample fully represented the initially identified target population, due to limited reporting on exclusions, missing data, or attrition. D6 and D7 were both judged as Yes in all studies, as SDB risk was assessed using previously validated and widely adopted screening questionnaires applied in a consistent manner within each study. D8 was rated as Yes across all studies, as the statistical methods used were consistent with the descriptive aims of prevalence-focused cross-sectional research. D9 was rated as No in all studies, as none reported response rates or described strategies to address potential non-response bias. Taken together, the predominance of Yes judgments in key methodological domains, alongside recurring Unclear or No ratings in domains related to sample representativeness and response handling, indicates a mixed risk-of-bias profile. On this basis, all included studies were considered to exhibit an overall moderate level of methodological quality. A detailed account of the domain-level assessments is provided in Table 7.

A detailed account of the domain-level assessments is provided in Table 7.

Following the domain-specific evaluation performed with the JBI Critical Appraisal Checklist—which indicated an overall moderate methodological quality with recurrent limitations in sample adequacy and response-rate reporting—the certainty of the evidence was further examined using the GRADE approach. This complementary assessment provided a broader perspective on the robustness of the findings, revealing a very low level of certainty for the prevalence of positive screening of SDB and for questionnaire items (within-tool correlates) and a low level of certainty for associations related to male gender and younger age. These results underscore the need for cautious interpretation of the available evidence. The findings of the GRADE are presented in Table 8.

## 4. Discussion

This systematic review found a wide range of positive screening for SDB among the orthodontic population. Among these, the nine investigations that employed the PSQ showed relatively consistent results, generally clustering within a narrow range despite differences in setting and sample size. By contrast, studies applying alternative tools such as the OSA-18, ESS-CHAD, or the SCR revealed much greater variability, with prevalence estimates ranging from as low as 1.2% to as high as 69%. The markedly lower prevalence observed with the OSA-18 may be explained by its design as a quality-of-life questionnaire rather than a direct screening instrument; its higher cut-off thresholds reduce its sensitivity for detecting milder or subclinical cases, and therefore underestimates high-risk prevalence [37].

On the other hand, the high prevalence detected with the SCR reflects its role as a structured clinical score specifically designed to maximize sensitivity in identifying children at risk for SDB, which may lead to overestimation when compared with standard questionnaires [16].

The prevalence of OSA in the pediatric population has shown considerable variability over time. Earlier community-based studies reported relatively low rates: Urschitz et al. (2010) [38] described a prevalence of approximately 2.8% among school-aged children (7–12 years); Brockmann et al. (2012) [39] estimated the prevalence of primary snoring to be around 6%; and the review by Kaditis et al. (2016) [40] summarized most studies as indicating a prevalence generally between 1 and 4%, with upper values close to 13%. In more recent years, however, higher figures have been documented: the meta-analysis by Zhang et al. (2024) [41] reported an overall prevalence of SDB of about 11% (95% CI 2–20%) in children and adolescents, while for preschool children these values are even bigger; the systematic review by Magnúsdóttir et al. (2024) [42] found estimates in preschool children ranging from 12.8% to 20.4%.

Within the dental field, Di Carlo et al. (2020) [43] identified a prevalence of approximately 9.7% of children at risk for SDB, as determined through the PSQ. Across the included studies, the proportion of orthodontic patients screening positive for SDB showed marked variability, ranging from as low as 1.2% to as high as 69%. Notably, five out of ten studies reported prevalence estimates of 20% or higher. Compared with estimates reported for the general pediatric population, these findings suggest a potentially higher burden of positive SDB screening in orthodontic cohorts; however, this observation should be interpreted with caution. The observed variability may reflect differences in study populations and selection factors, as individuals seeking orthodontic care frequently present craniofacial characteristics that have been associated with an increased risk of SDB, including maxillary constriction, mandibular retrognathia, and transverse discrepancies [44].

In the present review, as a secondary outcome, we identified a series of demographic characteristics that various studies have reported as statistically significant in relation to SDB. We found that male gender and younger age are often associated with a higher risk of SDB; this finding is largely confirmed by the most recent literature.

Recent evidence underscores that male sex is consistently associated with an increased prevalence and severity of OSA, with large cohort studies and systematic reviews confirming statistically significant differences between boys and girls [45,46].

Younger age also emerges as a critical factor, as children in the preschool range show an independently higher risk of developing moderate-to-severe OSA; this finding is largely confirmed by the recent literature [47].

From a craniofacial standpoint, transverse maxillary constriction and the presence of a high, narrow palate have been significantly associated with positive screening for SDB in orthodontic samples, consistent with biomechanical evidence and interventional studies supporting the role of rapid maxillary expansion in improving airway patency [48]. In addition, obesity and adenotonsillar hypertrophy remain robust and clinically relevant predictors of disease severity, as confirmed in recent pediatric cohorts where both factors significantly increased the odds of moderate-to-severe OSA [49,50].

Taken together, these findings reinforce the multifactorial nature of pediatric OSA and emphasize that risk stratification should integrate demographic, craniofacial, and clinical characteristics, rather than relying on a single domain.

In the context of SDB, the dentist—and more specifically the orthodontist—should play a pivotal role, not only as a fundamental figure in early screening but also in view of the positive impact that orthodontic interventions can exert in alleviating SDB-related symptoms [51].

The most recent studies clearly indicate an improvement in the AHI following treatment with rapid maxillary expansion, and the use of mandibular advancement devices also appears to exert a positive effect [52,53].

Nevertheless, awareness of healthcare professionals toward pediatric obstructive sleep apnea remains heterogeneous and, in many cases, insufficient. Recent findings among Italian pediatricians indicate substantial variability in knowledge and confidence regarding OSAS, with nearly 70% reporting consideration of oral and otorhinolaryngological features during examinations, yet marked differences in familiarity with diagnostic pathways and management guidelines [54]. Similar concerns are evident among dental professionals. In a large survey from Saudi Arabia, more than half of dentists acknowledged having received some education about OSA during undergraduate training, but fewer than 20% had ever diagnosed a case, and less than 8% had managed patients with oral appliances, reflecting a significant gap between theoretical knowledge and clinical practice [55]. Comparable results were observed in Iran, where general dentists and dental students demonstrated significantly lower knowledge and attitude scores than orthodontic residents, highlighting the absence of OSA in standard undergraduate curricula [56]. This trend is consistent with a European study showing that orthodontists, by virtue of their direct involvement with craniofacial growth and airway development, display comparatively higher awareness and readiness to intervene than general dentists [57]. Collectively, these findings underscore the urgent need for targeted education and training across pediatric and dental settings in order to enhance early recognition, referral, and multidisciplinary management of SDB in children.

### 4.1. Study Limitations

Despite the inclusion of studies with moderate methodological quality, certain limitations must be acknowledged as follows:

- All the studies included are cross-sectional; given the low to very low as assessed using the GRADE framework, the conclusions of this review should be interpreted with caution and do not allow for strong causal or clinical inferences.
- The orthodontic setting may have introduced selection bias, as this population could include a higher proportion of individuals with craniofacial features associated with SDB, potentially limiting the generalizability of the findings to the broader pediatric population.
- The use of different standardized questionnaires across studies may affect the overall risk estimates.
- The wide age range of the included patients may have introduced substantial heterogeneity and potentially affected the interpretation of the findings.
- The main outcome of the research is a probability of risk rather than a confirmed diagnosis of SDB, which at present can only be reliably obtained through polysomnography.

### 4.2. Future Directions

Looking forward, future research in this field would benefit from studies conducted directly within orthodontic settings, where patient selection and clinical conditions more closely reflect real-world practice. Harmonized diagnostic criteria and consistent screening protocols would allow for more comparable estimates across studies, reducing the current variability in reported prevalence. Moreover, the use of more robust and objective diagnostic methods, rather than relying solely on questionnaires or heterogeneous clinical indicators, will be essential to obtain more accurate estimates of SDB in children and adolescents. Collectively, these improvements would help clarify the true burden of SDB within orthodontic populations and support more informed clinical decision-making.

## 5. Conclusions

This systematic review suggests that SDB may represent a relevant concern within the orthodontic population. Based on the currently available evidence, which remains heterogeneous and primarily questionnaire-based:

- The proportion of orthodontic patients screening positive for SDB varied widely, with reported estimates ranging from very low to markedly high values.
- Males and preschool children appear to exhibit a potentially higher susceptibility to SDB;
- Several questionnaire-derived items, such as mouth breathing, nocturnal breathing difficulties, allergies, ADHD-related symptoms, and enuresis, were consistently associated with positive screening for SDB.

In light of the methodological heterogeneity and the predominant reliance on questionnaire-based screening, further investigations, employing standardized diagnostic frameworks and objective sleep assessments, are warranted to substantiate these observations and to more precisely delineate the actual prevalence of SDB within orthodontic populations.

## Figures and Tables

**Table 1:** Search strategy for each database.

Database	Search Strategy
Pubmed	((“sleep apnea syndromes”[MeSH Terms] OR “sleep apnea”[Title/Abstract] OR “obstructive sleep apnea”[Title/Abstract] OR “obstructive sleep apnoea”[Title/Abstract] OR “sleep-disordered breathing”[Title/Abstract] OR “sleep disordered breathing”[Title/Abstract] OR (“sleep”[Title/Abstract] AND “apnea”[Title/Abstract])) AND (“orthodontics”[MeSH Terms] OR “orthodontic*”[Title/Abstract] OR “orthodontics”[Title/Abstract] OR “orthodontal”[Title/Abstract]) AND (“diagnosis”[Subheading] OR diagnos*[Title/Abstract] OR screen*[Title/Abstract] OR “mass screening”[MeSH Terms] OR “mass screening”[Title/Abstract] OR (“early”[Title/Abstract] AND “detection”[Title/Abstract]))
Web of Science	TS = (“sleep disordered breathing” OR “sleep apnea”) AND TS = (orthodontic* OR orthodontial) AND TS = (screening OR diagnosis OR detection)
Scopus	TITLE-ABS-KEY (“sleep disordered breathing” OR “sleep apnea”) AND TITLE-ABS-KEY(orthodontic* OR orthodontics OR orthodontal) AND TITLE-ABS-KEY (screening OR diagnosis OR detection)
Embase	(‘sleep disordered breathing’ OR ‘sleep apnea’) AND (orthodontic* OR orthodontal OR orthodontics) AND (screening OR diagnosis OR detection)
Cochrane Library	(“sleep disordered breathing” OR “sleep apnea”) AND (orthodontic* OR orthodontics OR orthodontal) AND (screening OR diagnosis OR detection)

**Table 2:** PICO question.

PICO	
**Participant**	Children and adolescents (4–18 years) seeking orthodontic treatment or receiving orthodontic treatment.
**Intervention**	Screening for Sleep disordered Breathing through scientifically validated questionnaires.
**Comparison**	No group control.
**Outcome**	Prevalence of positive screening for SDB.

**Table 3:** General characteristics.

Author (Year)	Country	Study Setting	Study Design
Tsuda et al. (2011) [26]	Canada	Undergraduate program at the University of British Columbia	Cross-sectional
Rohra et al. (2018) [27]	USA	University Orthodontic Clinic, Case western reserve university, Cleveland	Cross-sectional
Abtahi et al. (2020) [36]	Canada/Saudi Arabia	University of Alberta clinic, private practices in Alberta.	Cross-sectional
Al Ehaideb et al. (2021) [28]	Saudi Arabia	Department of orthodontics at King Abdulaziz Dental Center, Riyadh.	Cross-sectional
Choong et al. (2022) [25]	Malaysia	Universiti Malaya (UM), Universiti Kebangsaan Malaysia (UKM), and Universiti Teknologi MARA (UiTM).	Cross-sectional Multicentric
Wellham et al. (2023) [29]	Australia	Private orthodontic practice, Perth	Cross-sectional
Orbach et al. (2023) [30]	Israel	Orthodontic Clinic at Hadassah Medical Center	Cross-sectional
Jost et al. (2024) [31]	Brazil/USA	Orthodontic clinic of Bauru Dental School	Cross-sectional
Kanwal et al. (2025) [32]	Pakistan	Orthodontic clinics at Aga Khan University Hospital	Cross-sectional
Niu et al. (2025) [33]	Denmark/Australia	Postgraduate Clinic of Orthodontics, Aarhus University	Cross-sectional
Nucci et al. (2025) [34]	Italy	Department of Orthodontics of the University of Catania	Cross-sectional
Storari et al. (2025) [35]	Italy	Department of Orthodontics, San Giovanni di Dio Hospital	Cross-sectional

**Table 4:** The primary outcome of the selected articles.

Author (Year)	Questionnaire	Sample Size	Mean Age (Age Range)	Gender M:F	% High Risk	% Low Risk
Tsuda et al. (2011) [26]	OSA-18	173	10.1 (8–12)	87 M 86 F	1.2%	98.8%
Rohra et al. (2018) [27]	PSQ	303	14.4 (9–17) y	152 M 151 F	7.3%	92.7%
Abtahi et al. (2020) [36]	PSQ	390	10.3 (5–16) y	173 M 217 F	10.8%	89.2%
Al Ehaideb et al. (2021) [28]	PSQ	285	14.1 (5–18) y	133 M 152 F	47.7%	52.3%
Choong et al. (2022) [25]	ESS-CHAD	105	14.3 (5–17) y	NS	29.5%	70.5%
Wellham et al. (2023) [29]	PSQ	1209	11 (6–18) y	561 M 647 F 1 NB	7.3%	92.7%
Orbach et al. (2023) [30]	PSQ	309	12 (6–17) y	146 M 163 F	10%	90%
Jost et al. (2024) [31]	PSQ	245	11.4 (5–18) y	115 M 130 F	34.3%	65.7%
Kanwal et al. (2025) [32]	PSQ	60	10 (7–12) y	30 M 30 F	20%	80%
Niu et al. (2025) [33]	PSQ	246	12.7 (6–16) y	94 M 152 F	6.5%	93.5%
Nucci et al. (2025) [34]	PSQ	364	12.2 (6–14) y	196 M 168 F	9.9%	90.1%
Storari et al. (2025) [35]	SCR	48	8.1 (5–12) y	17 M 31 F	69%	31%

M = male, F = female, NB = non binary, PSQ = Pediatric Sleep Questionnaire, SCR = Sleep Clinical Record, OSA-18 = Obstructive Sleep Apnea-18 questionnaire, ESS-CHAD = Epworth Sleepiness Scale for Children and Adolescents.

**Table 5:** The secondary outcome of the selected studies.

Questionnaire	Questionnaire Items (Within-Tool Correlates)	Studies Reporting Significant Association (*p* ≤ 0.05)
PSQ	Mouth breathing	Al Ehaideb et al. (2021) [28]
PSQ	Trouble breathing during sleep (snoring, pauses, labored breathing)	Al Ehaideb et al. (2021) [28]
PSQ	Allergy	Kanwal et al. (2025) [32]
PSQ	ADHD-related symptoms (inattention/hyperactivity)	Abtahi et al. (2020) [36]
PSQ	Enuresis	Abtahi et al. (2020) [36]
SCR	Mouth breathing	Storari et al. (2025) [35]
SCR	Narrow or high palatal vault (SCR morphological item)	Storari et al. (2025) [35]
OSA-18	No questionnaire-based risk factors reported	—
ESS-CHAD	No questionnaire-based risk factors reported	—

**Table 6:** Secondary outcome about demographic characteristics.

Risk Factors	Authors (Year)
Gender M	Al Ehaideb et al. (2021) [28]; Wellham et al. (2023) [29]; Orbach et al. (2023) [30]; Nucci et al. (2025) [34]
Younger age	Jost et al. (2024) [31]; Nucci et al. (2025) [34]

**Table 7:** Risk of bias assessment using the JBI Critical Appraisal Checklist for Studies Reporting Prevalence Data.

Author (Year)	D1	D2	D3	D4	D5	D6	D7	D8	D9	Overall
Tsuda (2011) [26]	yes	yes	unclear	yes	unclear	yes	yes	yes	no	Moderate
Rohra (2018) [27]	yes	yes	unclear	yes	unclear	yes	yes	yes	no	Moderate
Abtahi (2020) [36]	yes	yes	unclear	yes	unclear	yes	yes	yes	no	Moderate
Al Ehaideb (2021) [28]	yes	yes	no	yes	unclear	yes	yes	yes	no	Moderate
Choong (2022) [25]	yes	yes	unclear	yes	unclear	yes	yes	yes	no	Moderate
Wellham (2023) [29]	yes	yes	unclear	yes	unclear	yes	yes	yes	no	Moderate
Orbach (2023) [30]	yes	yes	unclear	yes	unclear	yes	yes	yes	no	Moderate
Jost (2024) [31]	yes	yes	yes	yes	unclear	yes	yes	yes	no	Moderate
Kanwal (2025) [32]	yes	yes	unclear	yes	unclear	yes	yes	yes	no	Moderate
Niu (2025) [33]	yes	yes	yes	yes	unclear	yes	yes	yes	no	Moderate
Nucci (2025) [34]	yes	yes	no	yes	unclear	yes	yes	yes	no	Moderate
Storari (2025) [35]	yes	unclear	no	yes	no	yes	yes	yes	no	Moderate

**Table 8:** GRADE certainty of evidence.

Outcome	Studies	Evidence Type	Starting Level	Risk of Bias	Inconsistency	Indirectness	Imprecision	Publication Bias	Upgrading Factors	Overall Certainty
Prevalence of positive SDB screening	[25,26,27,28,29,30,31,32,33,34,35,36]	Cross-sectional	low	Serious (−1): clinic-based cross-sectional designs, selection bias	Serious (−1): wide variability across studies and tools	Not serious (0): population and setting consistent with PICO	Serious (−1): heterogeneous sample sizes, no CIs	undetected	none	Very low
Questionnaire-based correlates of screen-positive status	[28,32,35,36]	Cross-sectional	low	Serious (−1): within-tool correlations, non-independent measures	Serious (−1): inconsistent associations across studies	Serious (−1): outcomes embedded within screening tools	Serious (−1): small samples, exploratory analyses	undetected	none	Very low
Male sex association with positive screening	[28,29,30,34]	Cross-sectional	low	Serious (−1): unadjusted analyses	Not serious (0)	Not serious (0)	Serious (−1): limited number of studies	undetected	none	Low
Younger age association with positive screening	[31,34]	Cross-sectional	low	Serious (−1): cross-sectional design	Not serious (0)	Not serious (0)	Serious (−1): small cohorts	undetected	none	Low

## Data Availability

The data presented in this study are available in the article.

## Supplementary Materials

The following supporting information can be downloaded at: https://www.mdpi.com/article/10.3390/jcm15020802/s1, Table S1: PRISMA 2020 checklist.
